# Supplementary material for: Identification of fatty acid amide hydrolase as a metastasis suppressor in breast cancer
Source: Nat Commun. 2023 May 30;14:3130. doi: 10.1038/s41467-023-38750-9 (PMC10229544; doi:10.1038/s41467-023-38750-9)
Supplement: Supplementary file 1 — Supplementary Information [file 41467_2023_38750_MOESM1_ESM.pdf]

SUPPLEMENTARY INFORMATION

Identification of fatty acid amide hydrolase as a metastasis suppressor in breast cancer

Isabel Tundidor [1,2], Marta Seijo-Vila [1,2], Sandra Blasco-Benito [1,2], María Rubert-Hernández [1,2], Sandra Adámez [1], Clara Andradas [3,4], Sara Manzano [5], Isabel Álvarez-López [5,6], Cristina Sarasqueta [7,8], María Villa-Morales [9,10], Carmen González-Lois [11], Esther Ramírez-Medina [12], Belén Almoguera [12], Antonio J. Sánchez-López [13,14], Laura Bindila [15], Sigrid Hamann [16], Norbert Arnold [16], Christoph Röcken [17], Ignacio Heras-Murillo [18], David Sancho [18], Gema Moreno-Bueno [19], María M. Caffarel [5,20], Manuel Guzmán [1,21], Cristina Sánchez [1,2*], and Eduardo Pérez-Gómez [1,2*].

[1]Department of Biochemistry and Molecular Biology, Complutense University, Madrid, Spain.

[2]Instituto de Investigación Hospital 12 de Octubre, Madrid, Spain.

[3]Brain Tumor Research Program, Telethon Kids Institute, Nedlands, WA, Australia.

[4]Centre for Child Health Research, University of Western Australia, Nedlands, WA, Australia.

[5]Breast Cancer Group, Oncology Area, Biodonostia Health Research Institute, San Sebastián, Spain.

[6]Gipuzkoa Cancer Unit, OSI Donostialdea - Onkologikoa Foundation, San Sebastian, Spain.

[7]Unit of Information and Healthcare Results, OSI Donostialdea, Biodonostia Health Research Institute, San Sebastián, Spain.

[8]Methodological Support Unit, Biodonostia Health Research Institute, San Sebastián, Spain.

[9]Centro de Biología Molecular Severo Ochoa (CBMSO) (CSIC-UAM), Madrid, Spain.

[10]Department of Biology, Autonomous University of Madrid, Madrid, Spain.

[11]Department of Pathology, Hospital Universitario Puerta de Hierro, Majadahonda, Madrid, Spain.

[12]Department of Obstetrics & Gynecology, Hospital Universitario Puerta de Hierro, Majadahonda, Madrid, Spain.

[13]Biobank Hospital Universitario Puerta de Hierro Majadahonda, Madrid, Spain.

[14]Instituto de Investigación Sanitaria Puerta de Hierro-Segovia de Arana (IDIPHISA), Madrid, Spain.

[15]Clinical Lipidomics Unit, Institute of Physiological Chemistry, University Medical Center, Mainz, Germany.

[16]Department of Gynecology and Obstetrics, University Hospital Schleswig-Holstein, Kiel, Germany.

[17]Institute of Pathology, University Hospital Schleswig-Holstein, Kiel, Germany.

[18]Immunobiology Laboratory, Centro Nacional de Investigaciones Cardiovasculares (CNIC), Madrid, Spain.

[19]MD Anderson International Foundation; Instituto de Investigaciones Biomédicas 'Alberto Sols' (CSIC-UAM); Department of Biochemistry, Autonomous University of Madrid; Instituto de Investigación Hospital Universitario La Paz (IdiPaz); Centro de Investigación Biomédica en Red de Cáncer (CIBERONC); Madrid, Spain.

[20]Ikerbasque - Basque Foundation for Science, Bilbao, Spain.

[21]Instituto Ramón y Cajal de Investigación Sanitaria y Centro de Investigación Biomédica en Red de Enfermedades Neurodegenerativas (CIBERNED), Madrid, Spain.

*To whom correspondence should be addressed:

Eduardo Pérez-Gómez, PhD
Department of Biochemistry and Molecular Biology
Instituto de Investigación Hospital 12 de Octubre
Complutense University,
Madrid 28040,
Spain
Email: eduperez@ucm.es

Cristina Sánchez, PhD
Department of Biochemistry and Molecular Biology
Instituto de Investigación Hospital 12 de Octubre
Complutense University,
Madrid 28040,
Spain
Email: macsanch@ucm.es

This Supplementary Information file includes:
- Supplementary Figures 1, 2, 3, 4, 5, 6, 7, 8, 9, 10, 11, 12 and 13
- Supplementary Data legends 1, 2, 3

Supplementary Figure 1

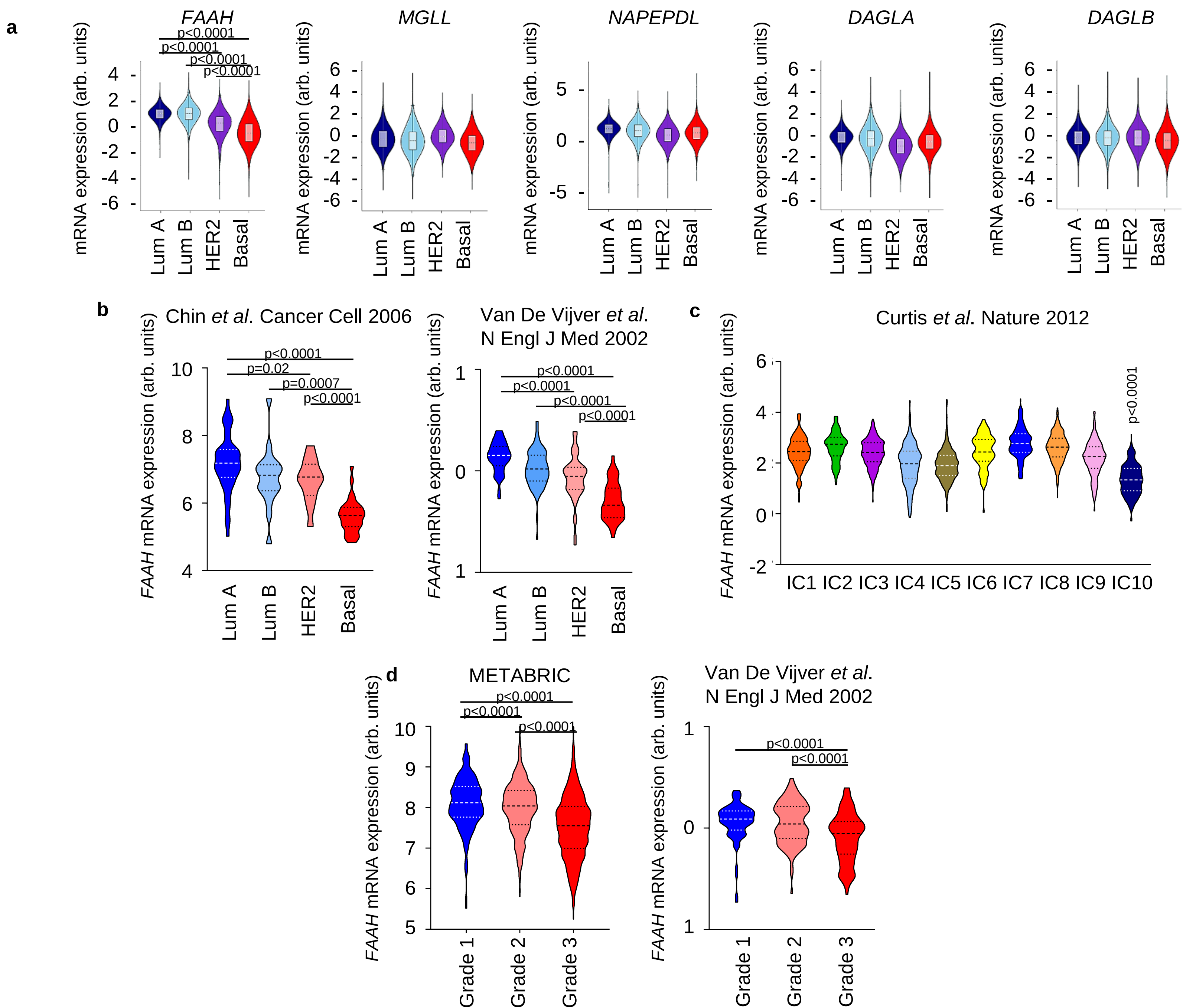

Supplementary Figure 1. FAAH is highly expressed in differentiated breast tumors. **a** Relative mRNA expression of the main enzymes involved in the biosynthesis (*NAPEPLD*, *DAGLA*, *DAGLB*) and degradation (*FAAH*, *MGLL*) of endocannabinoids along the four molecular subtypes of BC according to the bc-GenExMiner website. The colored box indicates the median and 25–75th percentile range, and the whiskers extend from the ends of the box to the smallest and largest data values. **b** Relative *FAAH* mRNA expression along the four molecular subtypes of BC according to the datasets published in NKI-295 and E-TABM-158 [64]. **c** Relative *FAAH* mRNA expression along the prognostic IC 1-10 signatures established by the METABRIC dataset. IC10 group is significantly different from the rest with a $p < 0.001$. **d** Relative *FAAH* mRNA expression in BC samples grouped by tumor grade according to the dataset published in METABRIC dataset and NKI-295. Data were analyzed by 1-way ANOVA with post Tukey's multiple comparison test. Source data are provided as a Source Data file.

Supplementary Figure 2

a

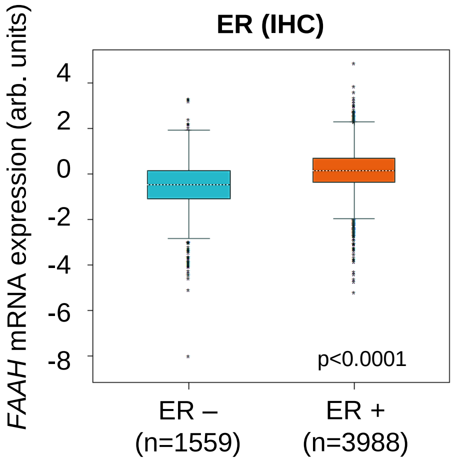

b

Database entry	ER+ (n)	ER- (n)	Fold Change	T-Statistic	P-Value
E-TABM-158	75	43	1.821	5.055	1.23×10^{-6}
GSE2034	209	77	1.597	6.301	3.30×10^{-9}
GSE12276	57	42	2.051	6.113	2.37×10^{-8}
TCGA	225	87	1.729	9.992	1.46×10^{-18}
GSE18728	32	29	1.721	5.025	3.72×10^{-6}
GSE7390	102	56	1.984	6.454	4.02×10^{-9}
EGAS00000000083	1156	352	1.787	21.396	4.02×10^{-72}
GSE20685	204	123	1.615	10.390	4.02×10^{-22}
GSE3744	15	24	2.647	4.424	1.13×10^{-4}
GSE5460	45	50	2.120	6.208	9.45×10^{-9}
GSE32646	71	44	1.891	4.481	1.31×10^{-5}

c

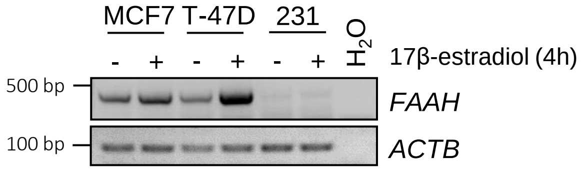

d

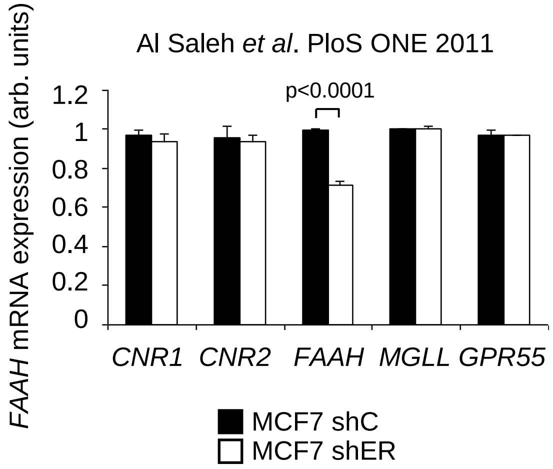

e

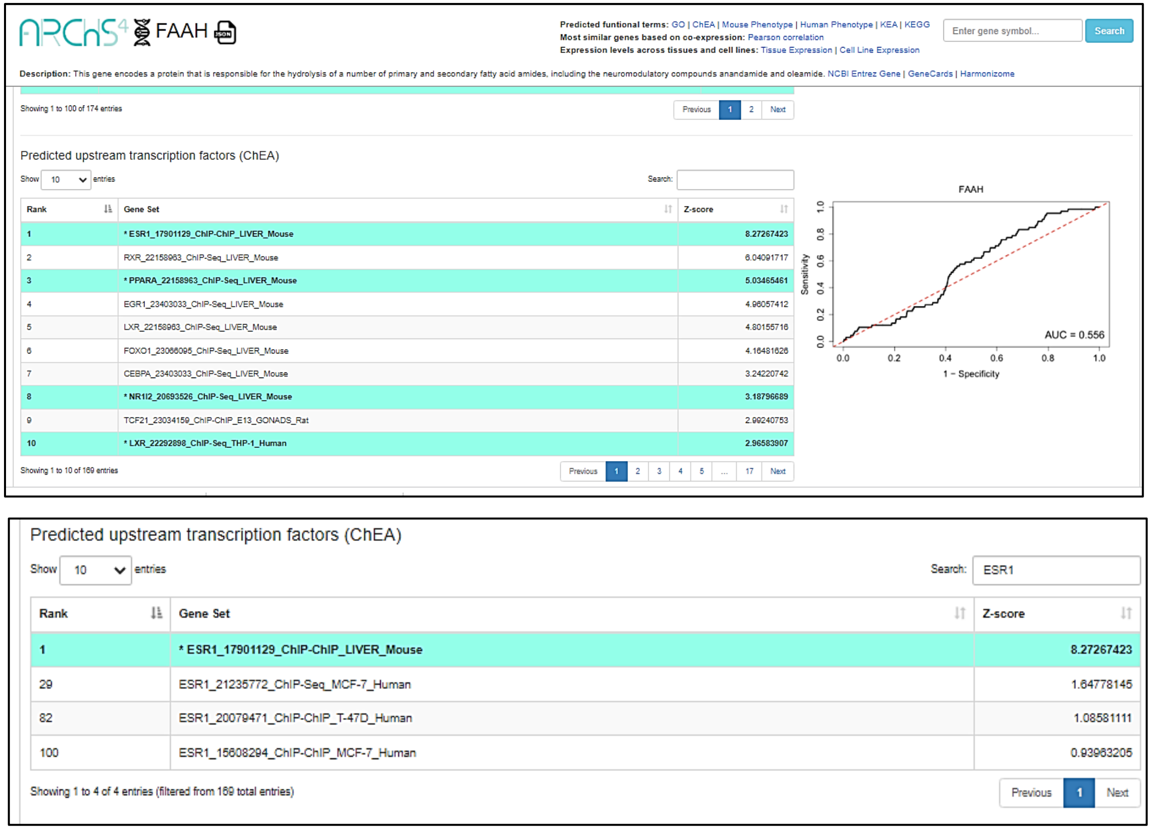

Supplementary Figure 2. *FAAH* mRNA expression is strongly associated with ER+ status in breast tumors. **a, b** Relative *FAAH* mRNA expression in BC samples grouped by their ER status according to the bc-GenExMiner website (a) and several other databases (b). The colored box in (a) indicates the median and 25–75th percentile range, and the whiskers extend from the ends of the box to the smallest and largest data values. Data were analyzed by 2-tailed Student's t-test (a, b). **c** Representative end-point RT-PCR analysis of *FAAH* mRNA expression in luminal BC cell lines after treatment with 10 nM 17β-estradiol. The ER- cell line MDA-MB-231 was used as a negative control. β-Actin (*ACTB*) was used as normalization control. n=3 biological replicates. **d** Relative mRNA expression of several ECS components in ER-silenced MCF7 cells according to the dataset GSE27473. Data were analyzed by 2-tailed Student's t-test. **e** Transcription factors binding the *FAAH* gene promoter according to the CHEA Transcription Factor Targets dataset. Source data are provided as a Source Data file.

Supplementary Figure 3

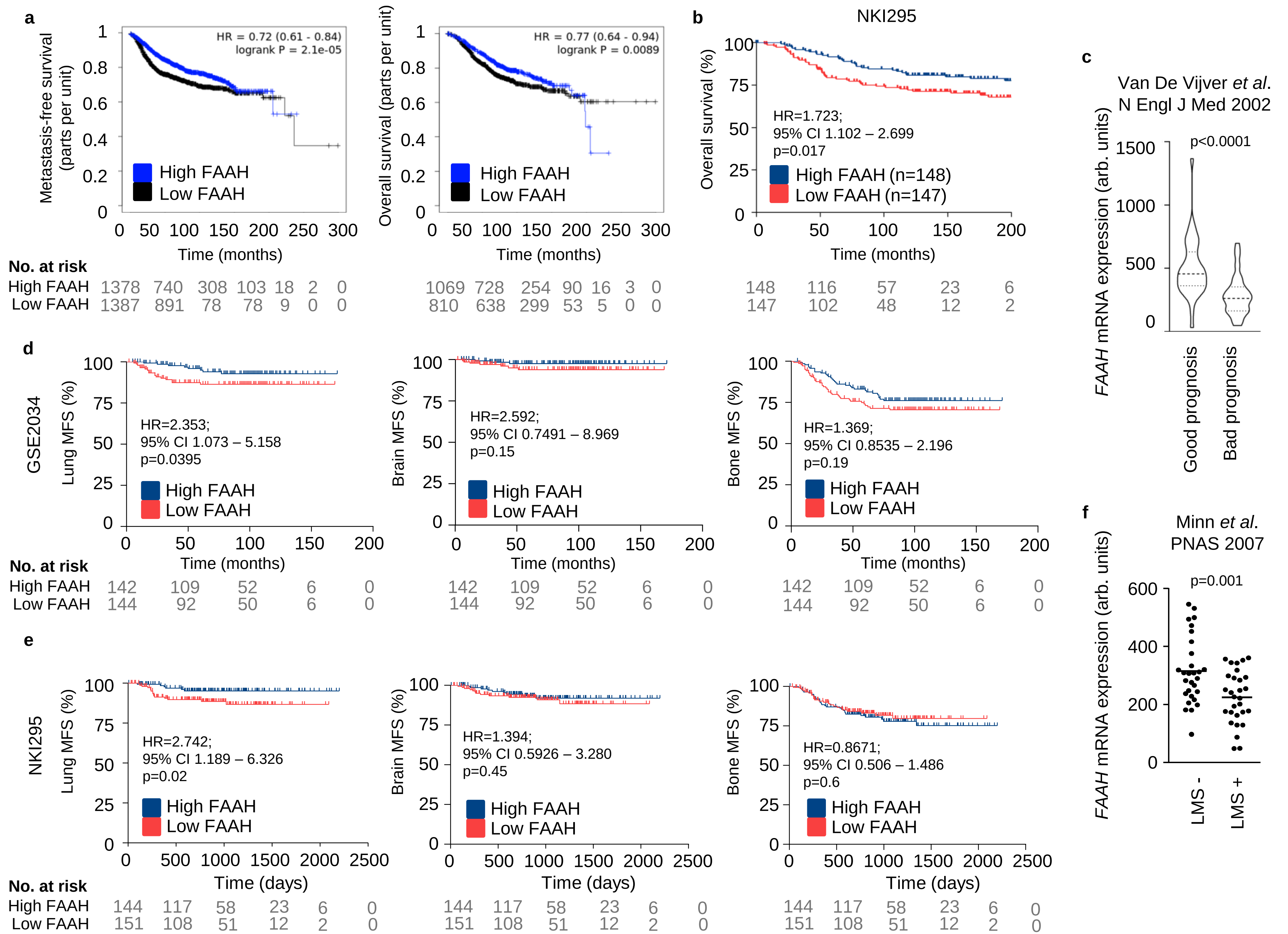

Supplementary Figure 3. Low tumor *FAAH* mRNA expression is associated with poor patient prognosis. **a, b** Kaplan-Meier curves for metastasis-free (a, left panel) and overall survival (a, right panel, and b) in BC samples with high and low *FAAH* mRNA expression obtained through the KM plotter website (a) and from the NKI-295 dataset (b). **c** Relative *FAAH* mRNA expression in BC samples classified according to the prognosis signature established by NKI-295. Data were analyzed by 2-tailed Student's t-test. **d**, **e** Kaplan-Meier curves for lung, brain and bone metastasis-free survival (MFS) in BC patients with high and low *FAAH* mRNA expression according to the datasets NKI-295 and GSE2034. Kaplan-Meier curves were statistically compared by the logrank test. **f** Relative *FAAH* mRNA expression in BC samples classified according to the lung metastasis signature (LMS). Data were analyzed by 2-tailed Student's t-test. Source data are provided as a Source Data file.

Supplementary Figure 4

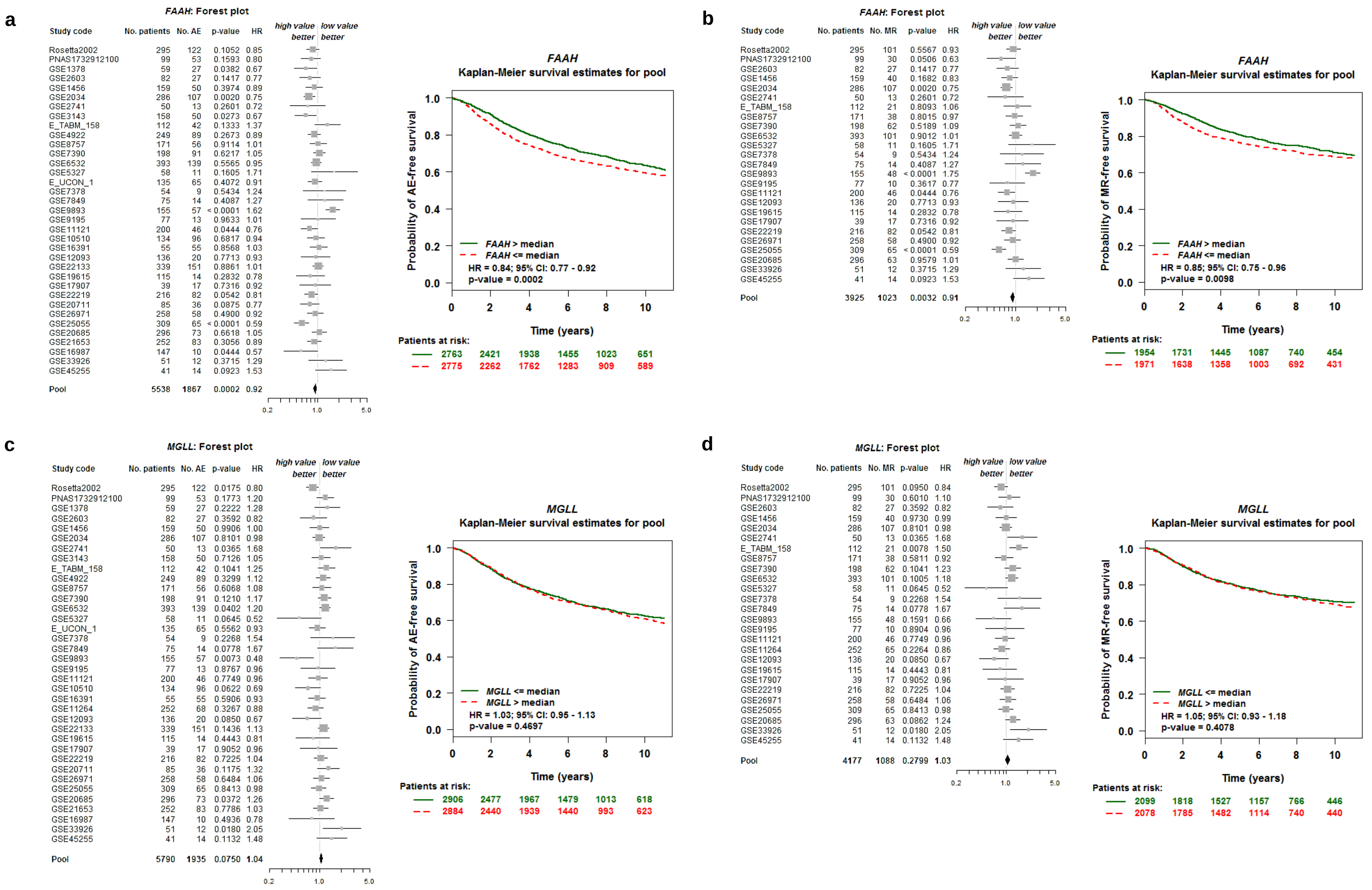

Supplementary Figure 4. Low tumor *FAAH* (and not *MGLL*) expression is associated with poor patient prognosis. **a-d** On the left of each panel, forest plots illustrating univariate Cox analysis of *FAAH* (a, b) or *MGLL* (c, d) mRNA expression in BC samples and their risk association with any event of relapse (AE) (a, c) or metastatic relapse (MR) (b, d) according to the bc-GenExMiner website. On the right, Kaplan-Meier curves representing data from the forest plots. Kaplan-Meier curves were statistically compared by the logrank test.

Supplementary Figure 5

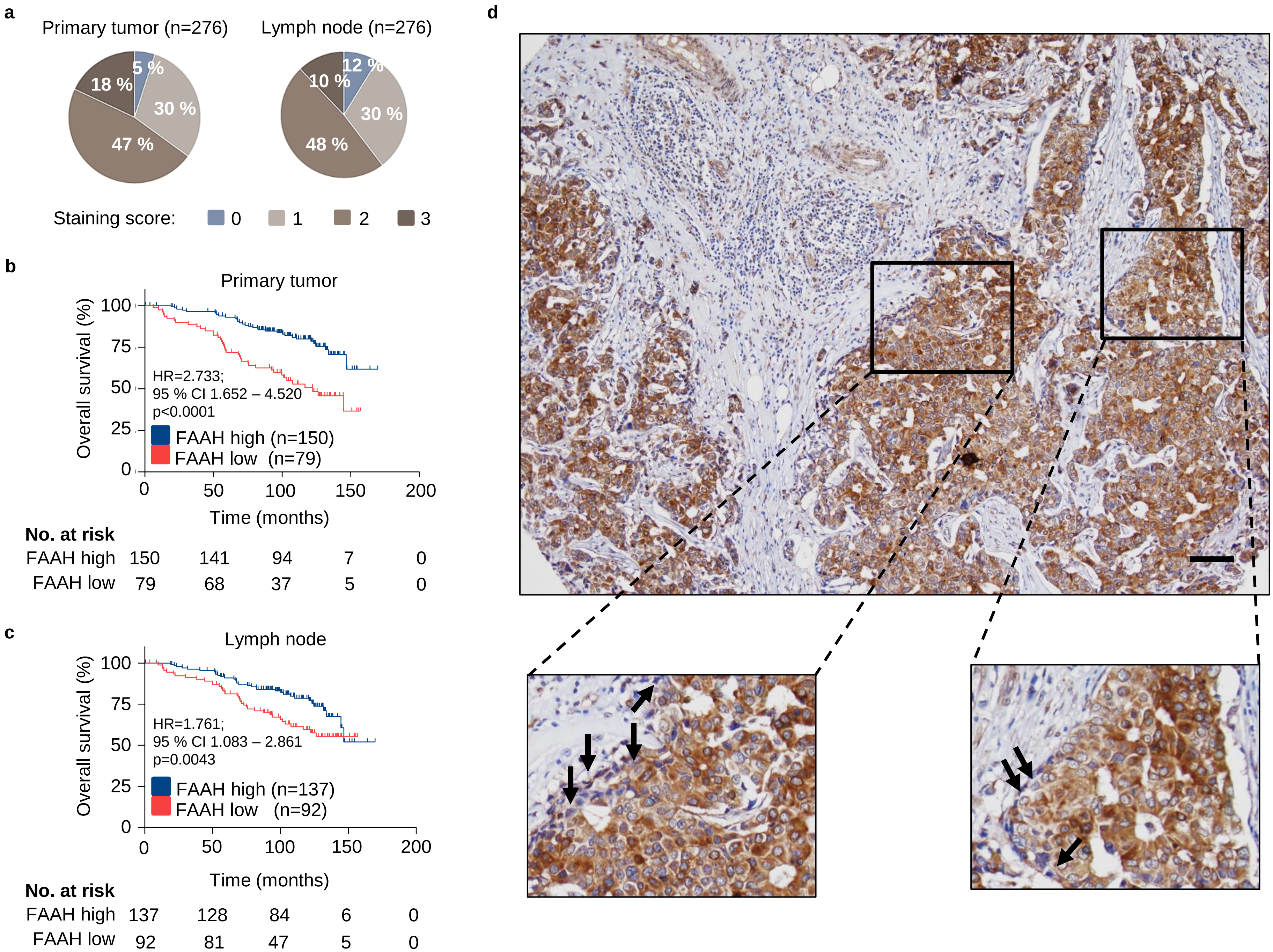

Supplementary Figure 5. FAAH expression is frequently downregulated during metastatic progression in luminal BC. **a** Pie charts representing FAAH expression in samples of primary tumor and LN metastasis in the BC samples included in TMA #2. **b, c** Kaplan-Meier curves for overall survival from patients included in TMA #2 according to their levels of FAAH expression in the primary tumor (b) and in the LN (c). Kaplan-Meier curves were statistically compared by the logrank test. **d** Representative image (n=2 biological replicates) and detail magnification of tumor samples from TMA #2. Arrows indicate cells with lower FAAH expression at the invasion front. Scale bar = 250 µm. Source data are provided as a Source Data file.

Supplementary Figure 6

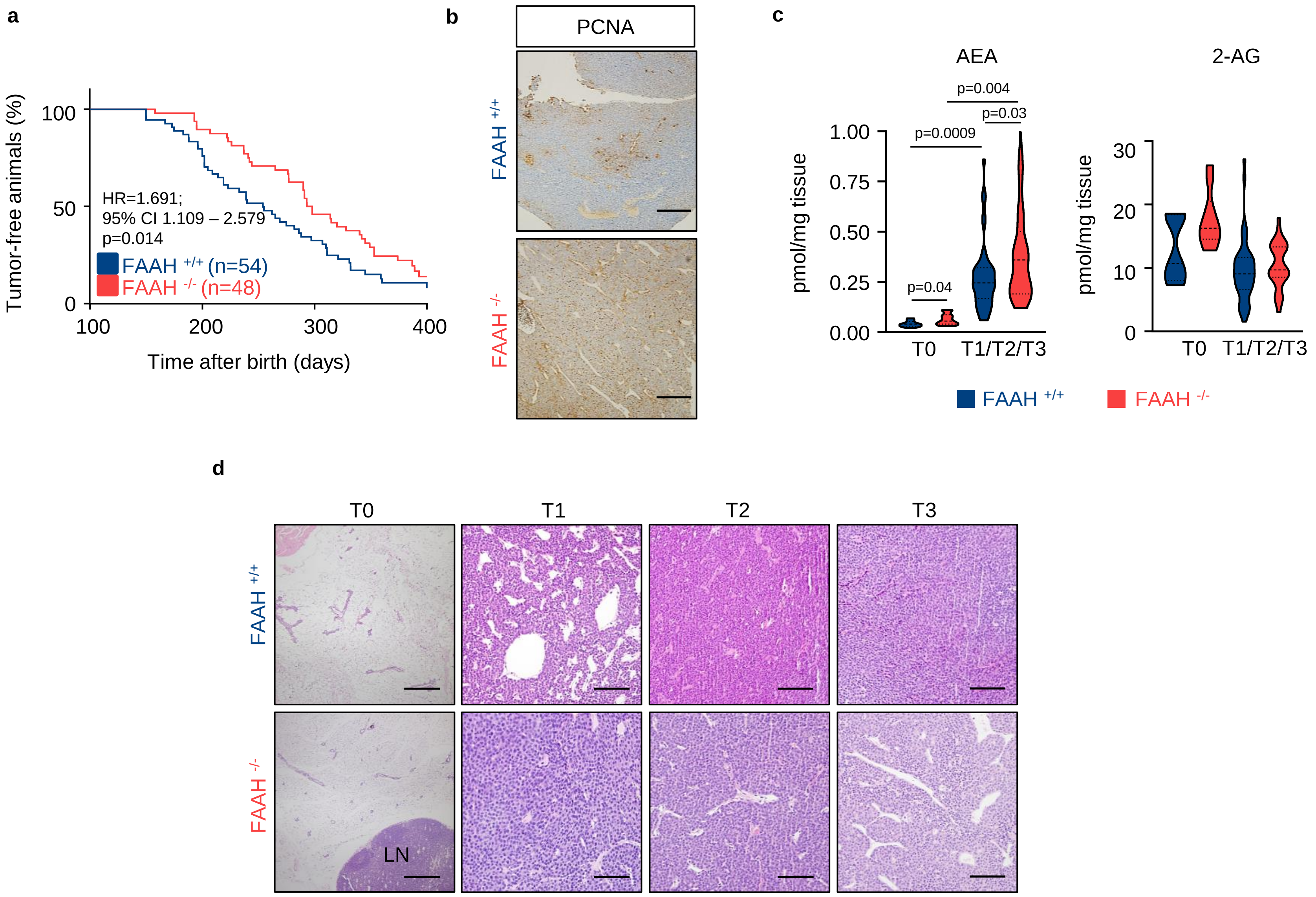

Supplementary Figure 6. FAAH genetic silencing promotes the formation of more aggressive breast tumors in mice. **a** Kaplan-Meier curves for tumor onset in MMTV-neu:FAAH $^{+/+}$ and FAAH $^{-/-}$ mice. Kaplan-Meier curves were statistically compared by the logrank test. **b** Representative IHC analysis of proliferating cell nuclear antigen (PCNA) in MMTV-neu-derived tumors (n=3 biological replicates). Scale bar = 250 µm. **c, d** Analysis of AEA and 2-AG levels (c) and representative H&E images of MMTV-neu-derived tumors (n>10 biological replicates) at different time points (T1, T2 and T3) and pre-lesion mammary glands (T0) (d). Data were analyzed by 2-tailed Student's t-test. LN = lymph node. Scale bar = 250 µm. Source data are provided as a Source Data file.

Supplementary Figure 7

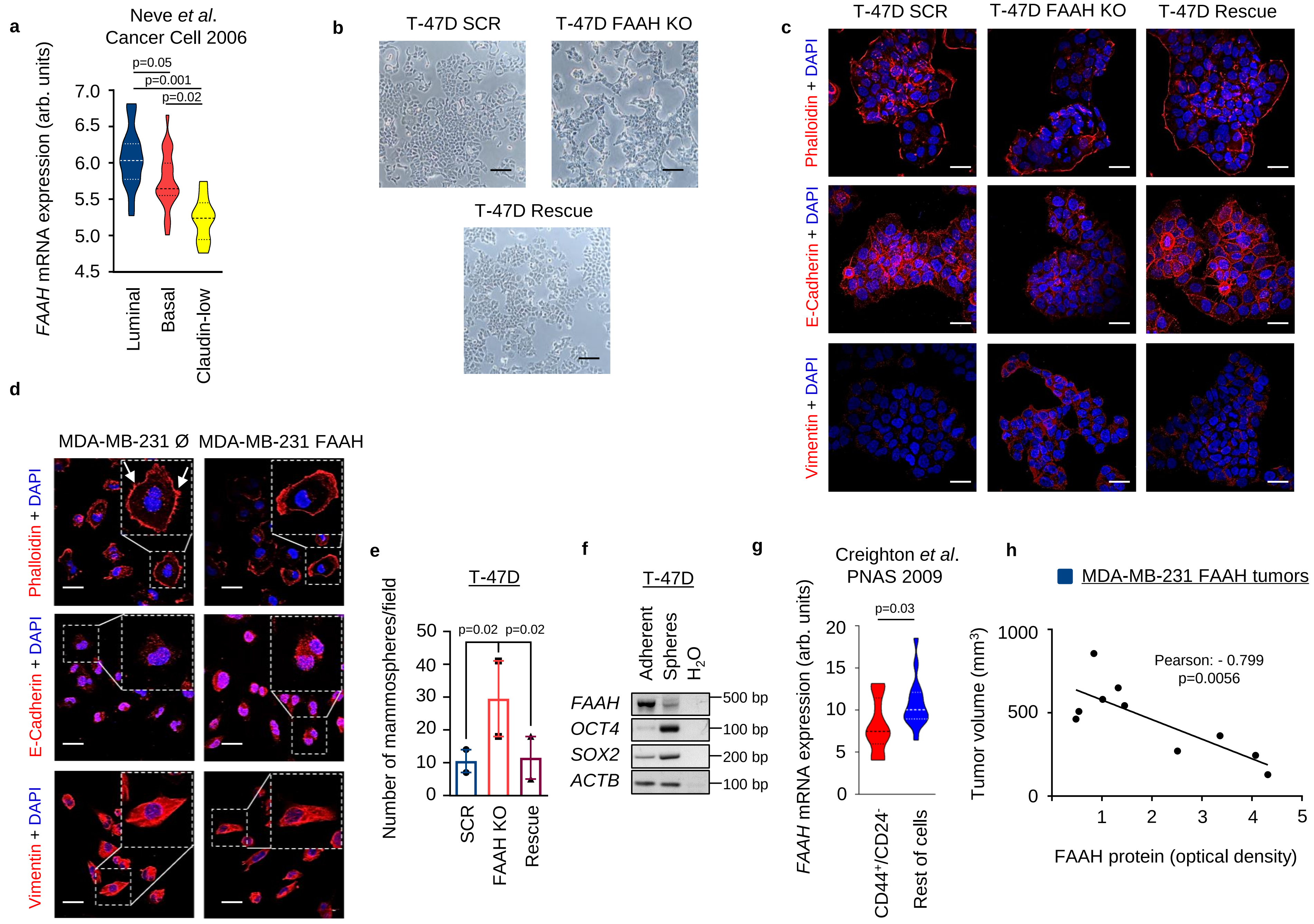

Supplementary Figure 7. FAAH regulates pro-oncogenic features of BC cells *in vitro* and *in vivo*. **a** *FAAH* mRNA expression in luminal, basal, and claudin-low BC cell lines according to the dataset GSE69017. Data were analyzed by 1-way ANOVA with post Tukey's multiple comparison test. **b** Representative bright-field images (n=3) showing morphologic variations of FAAH-modulated T-47D cells. Scale bar = 100 µm. **c, d** Immunofluorescence staining of cytoskeleton (phalloidin) and EMT markers (E-cadherin and vimentin) in FAAH-modulated T-47D (c) and MDA-MB-231 cells (d). Cell nuclei are in blue. Scale bar = 20 µm. n=3 biological replicates. **e** Effects of FAAH modulation on mammosphere formation by T-47D cells. Data are presented as mean values +/- SEM of n=3 biological replicates and were analyzed by 1-way ANOVA with post Tukey's multiple comparison test. **f** End-point RT-PCR analysis of *FAAH* and pluripotency markers (*OCT4*, *SOX2*) mRNA expression in a spheroid culture *vs*. an adherent culture of T-47D cells. β-Actin (*ACTB*) was used as normalization control. n=3 biological replicates. **g** Relative *FAAH* mRNA expression in BC samples sorted by their expression of CD44 and CD24 surface markers according to the GSE7515. Data were analyzed by 2-tailed Student's t-test. **h** Linear regression analysis representing tumor size *vs*. FAAH protein levels in tumors derived from FAAH-overexpressing MDA-MB-231 xenografts, as determined by densitometric quantification of WB analysis. Source data are provided as a Source Data file.

Supplementary Figure 8

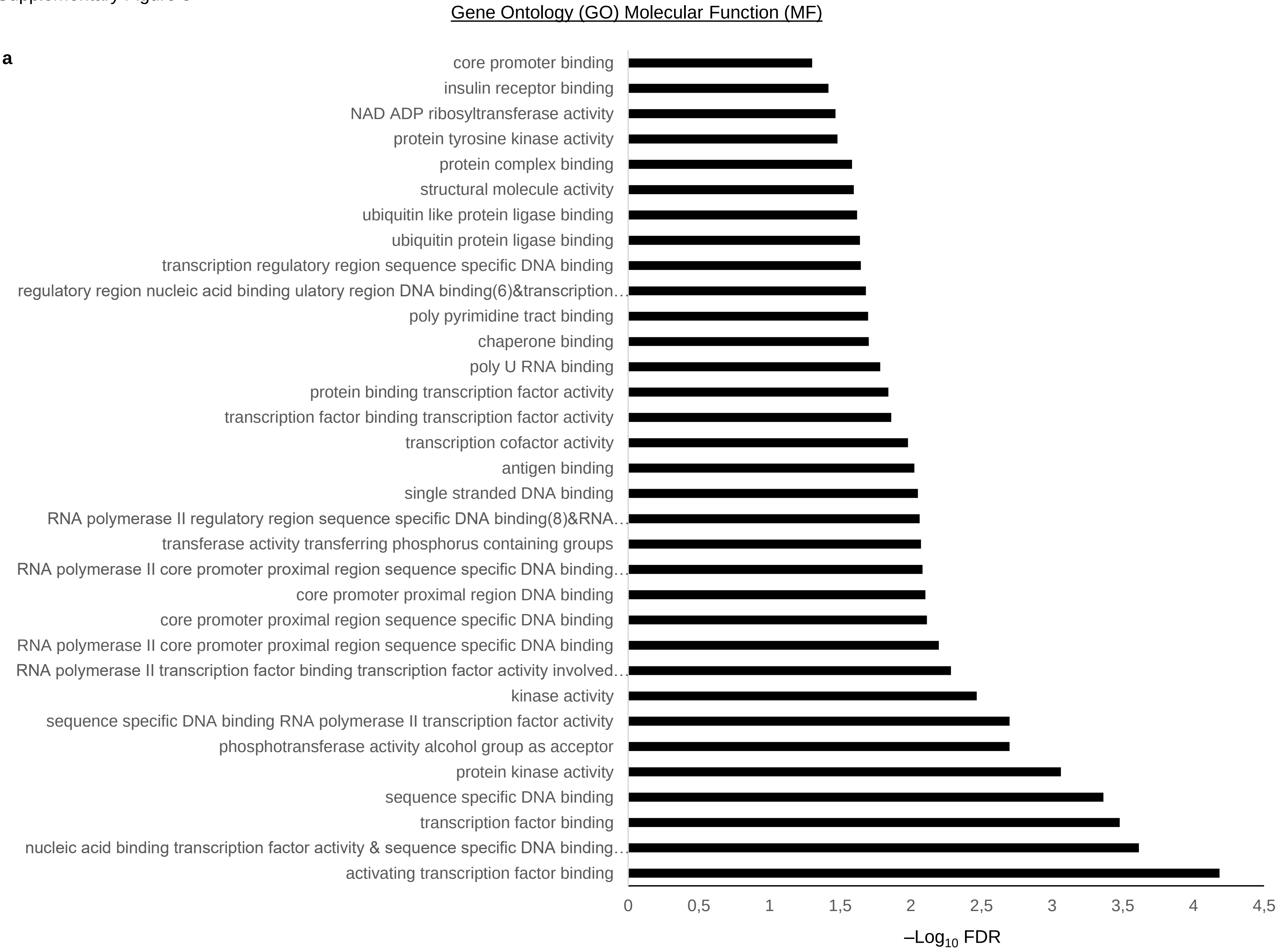

Supplementary Figure 8

Gene Ontology (GO) Biological Process (BP)

b

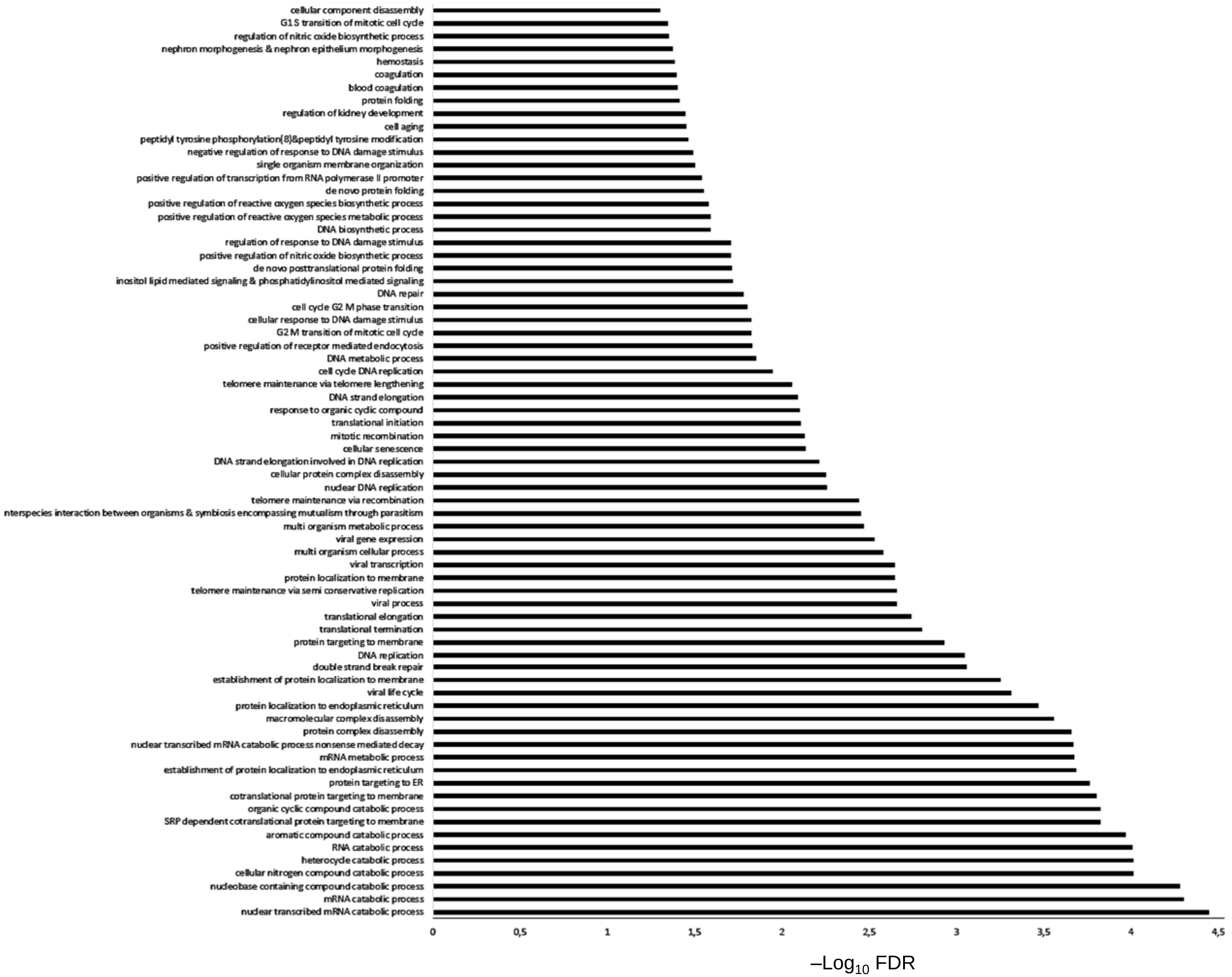

Supplementary Figure 8

Kyoto Encyclopedia of Genes and Genomes Pathways

c

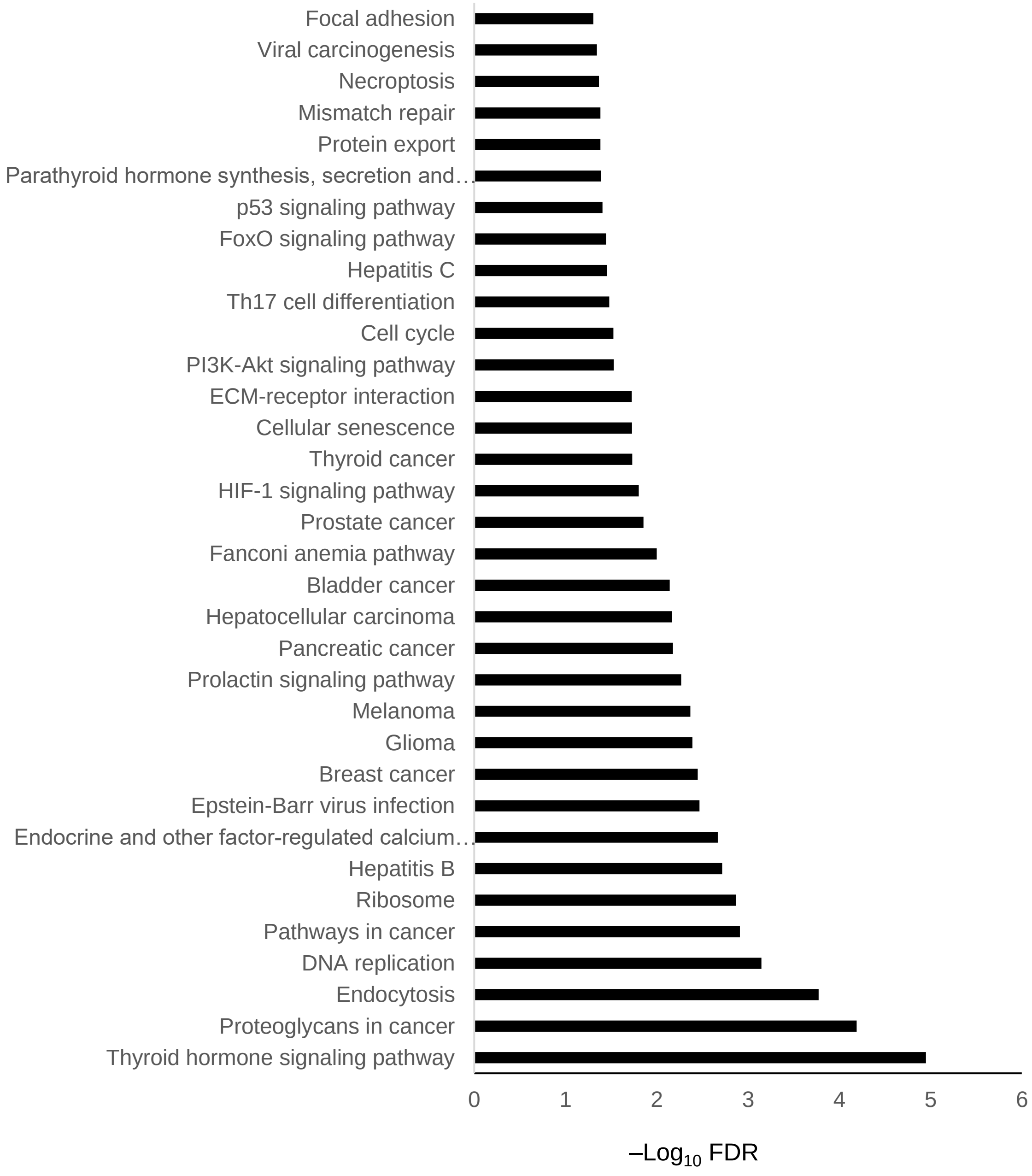

Supplementary Figure 8

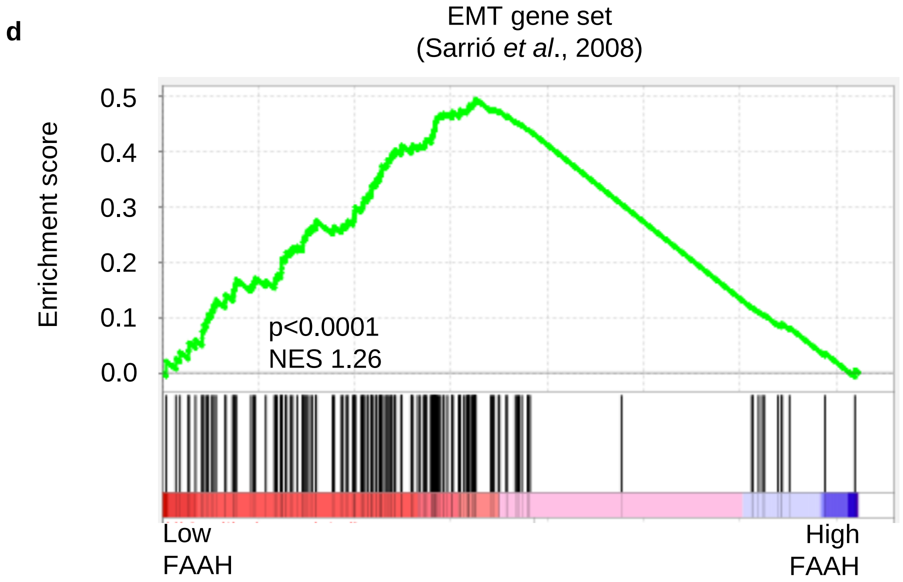

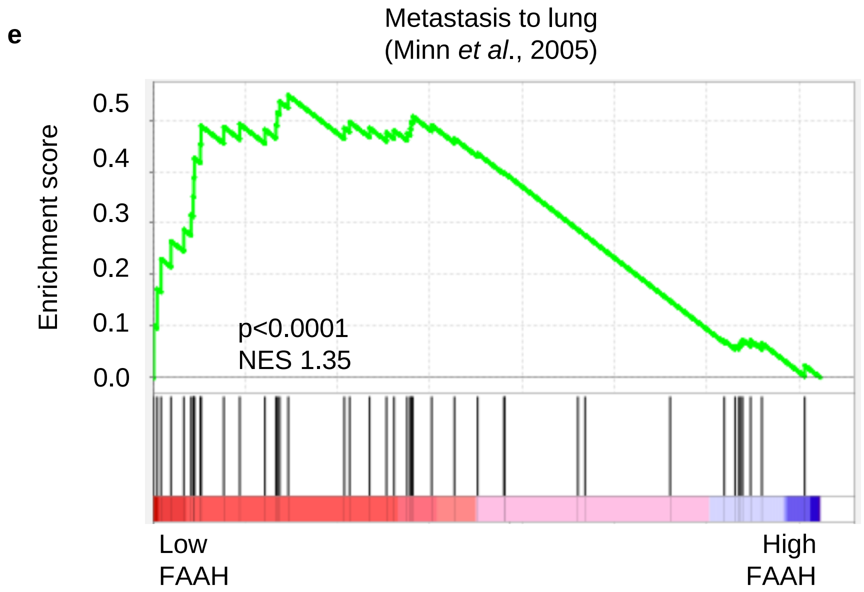

Supplementary Figure 8. Transcriptomic analyses in FAAH-modulated T-47D cells. **a-c** Bar graphs representing GO Molecular Function (MF) (a), Biological Processes (BP) (b), and Kyoto Encyclopedia of Genes and Genomes Pathways (KEGG) (c) analysis of DEGs in T-47D FAAH KO cells. **d**, **e** Gene Set Enrichment Analysis (GSEA) of DEGs in T-47D FAAH KO cells.

Supplementary Figure 9

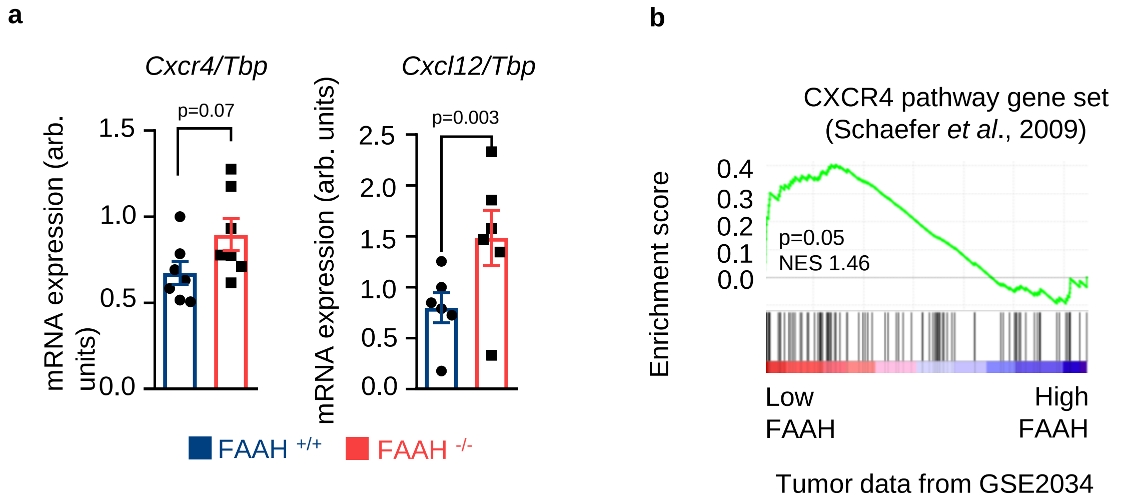

Supplementary Figure 9. FAAH and CXCR4/CXCL12 levels are inversely associated in mouse and human breast tumors. **a** Quantitative RT-PCR analysis of *Cxcr4* and *Cxcl12* mRNA levels in MMTV-neu FAAH $^{+/+}$ and FAAH $^{-/-}$ mice-derived tumors. Data are presented as mean values +/- SEM of n=7 biological replicates and were analyzed by 2-tailed Student's t-test. **b** Gene Set Enrichment Analysis (GSEA) showing an activation of the CXCR4 pathway in low FAAH-expressing BC samples from dataset GSE2034. Source data are provided as a Source Data file.

Supplementary Figure 10

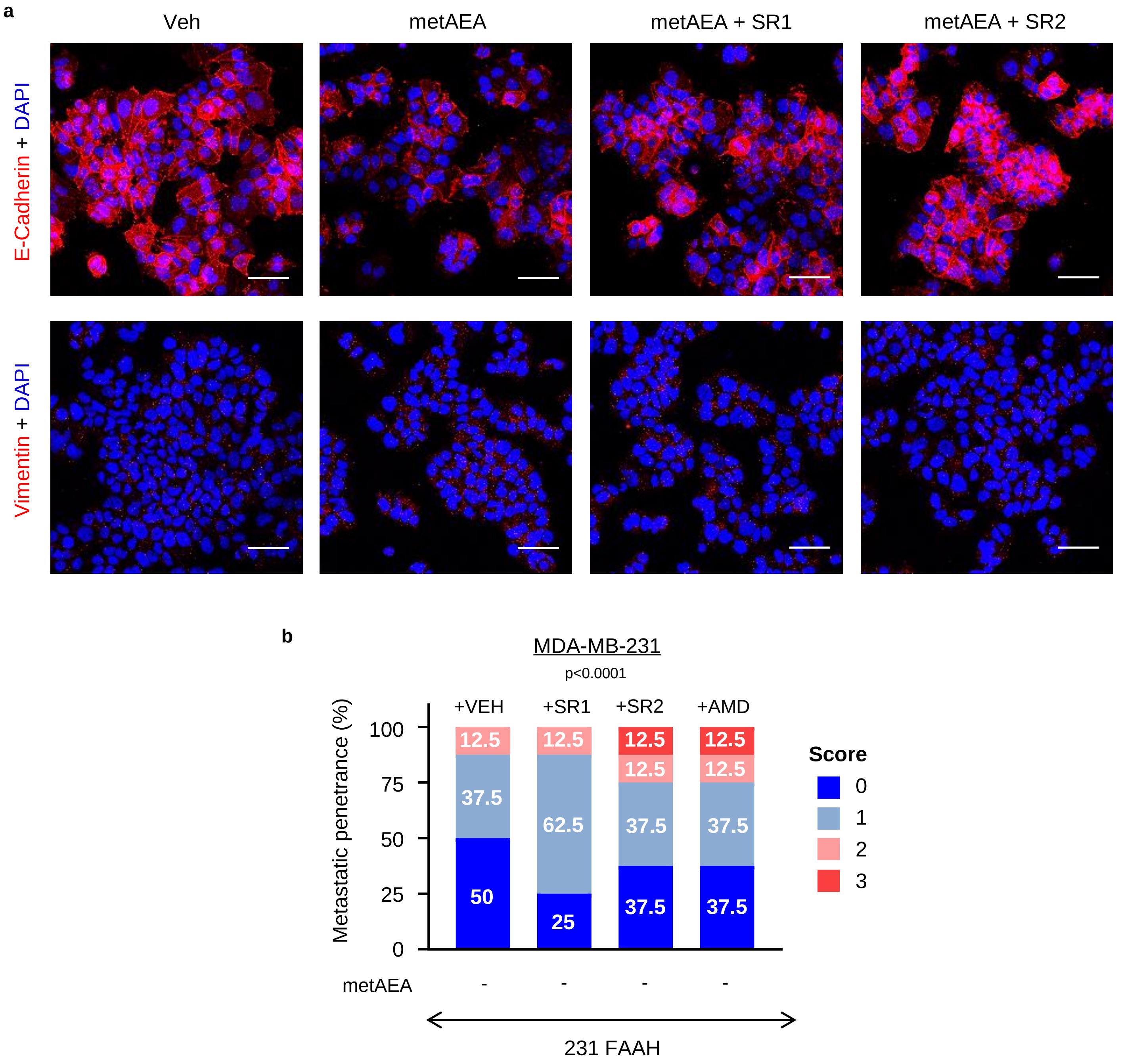

Supplementary Figure 10. **a** Immunofluorescence staining of EMT markers (E-cadherin and vimentin) in T-47D cells after a 4-week exposure to metAEA (1 nM) alone or in combination with SR1 or SR2 (1 μM). Cell nuclei are in blue. Scale bar = 20 μm. n=3 biological replicates. **b** Lung metastatic damage caused by FAAH-overexpressing MDA-MB-231 cells in immunocompromised mice treated with vehicle or SR1, SR2 and AMD3100 (2.5 mg/kg). Metastatic damage was monitored with an IVIS system and categorized into four scores depending on bioluminescence values. Pearson's chi-squared test was used for statistical analysis. Source data are provided as a Source Data file.

Supplementary Figure 11

	TMA #1	TMA #2
	n (%)	n (%)
**Age**		
<50	164/623 (26.3)	91/293 (31.0)
>50	459/623 (73.7)	202/293 (69.0)
**Histological Grade**		
1	74/598 (12.4)	39/283 (13.8)
2	371/598 (62.0)	165/283 (58.3)
3 and 4	153/598 (25.6)	79/283 (27.9)
**T-category**		
T1	365/572 (63.8)	86/293 (29.4)
T2	166/572 (29.0)	182/293 (62.1)
T3 and T4	41/572 (7.2)	25/293 (8.5)
**N-category**		
Negative	378/573 (66.0)	0/293 (0.0)
Positive	195/573 (34.0)	293/293 (100)
**M-category**		
Negative	414/430 (96.3)	222/287 (77.4)
Positive	16/430 (3.7)	65/287 (22.6)
**ER expression**		
Negative	466/608 (76.6)	0/293 (0.0)
Positive	142/608 (33.4)	293/293 (100)
**PR expression**		
Negative	393/508 (77.4)	47/293 (16.0)
Positive	215/508 (32.6)	246/293 (84.0)
**HER2 amplification**		
Negative	350/500 (70.0)	131/159 (82.4)
Positive	150/500 (30.0)	28/159 (17.6)
**Histological subtype**		
IDC	519/620 (83.7)	240/289 (83.0)
ILC	73/620 (11.8)	44/289 (15.2)
Others	28/620 (4.5)	5/289 (1.8)

Supplementary Figure 11. Summary of clinical, pathological and immunohistochemical features in the diagnosis of breast carcinomas included in TMA #1 and TMA #2. T-category: Primary tumor size; M-category: Tumor spreading to distant organs; N-category: lymph nodes tumor infiltration; ER: Estrogen receptor; PR: Progesterone receptor; IDC: Invasive ductal carcinoma; ILC: Invasive lobular carcinoma.

Supplementary Figure 12

GATING STRATEGY FOR FLOW CYTOMETRIC DETECTION OF CXCR4 IN HUMAN BC CELL LINES

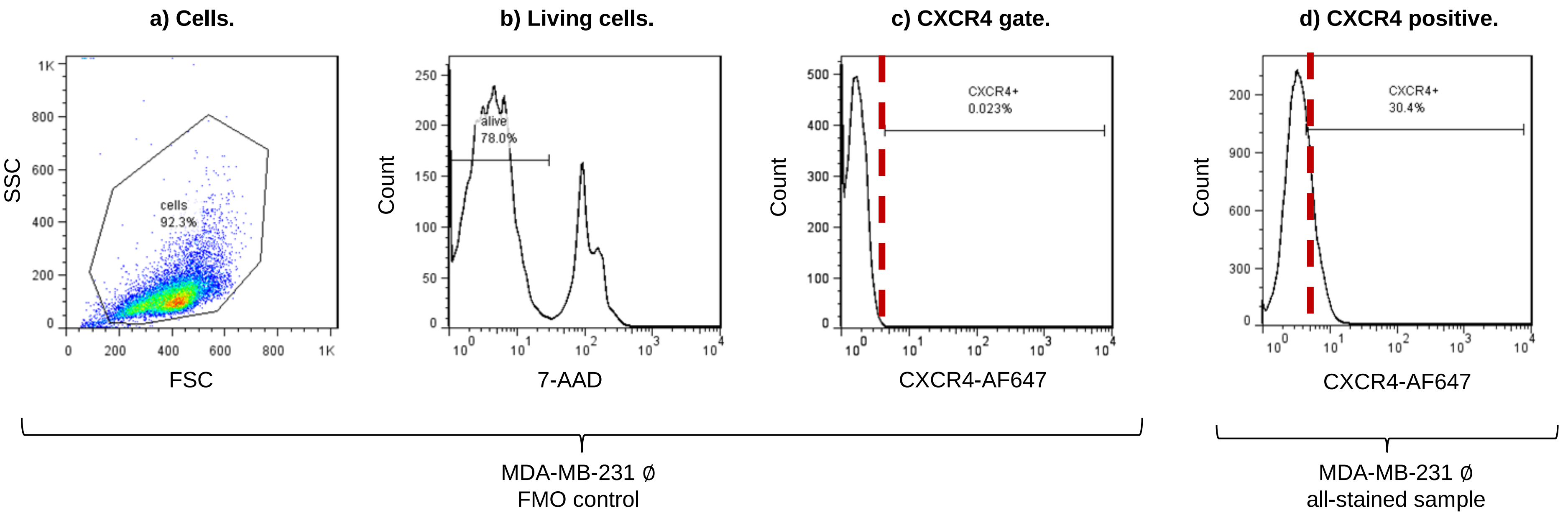

Supplementary Figure 12. Gating strategy used to determine the % of CXCR4 positivity in FAAH-modulated human BC cell lines. Gating strategy was always performed in fluorescence-minus-one (FMO) controls, which are samples stained with all the fluorophores except for CXCR4. There was a specific FMO control for each sample. **a** First, cells were separated from cell debris based on their size (FSC) and granularity (SSC). **b** 7-amino actinomycin D (7-AAD) was used as the live/dead discriminator. 7-AAD positive (dead) cells were excluded from the analysis. **c** The fluorescent signal of the remaining (7-AAD negative, alive) cells in the FMO was used to set the upper boundary for background signal and thus to identify and gate positive populations for CXCR4. **d** The gating strategy described in a-c was applied to the all-stained sample to establish the % of CXCR4 positive cells.

Supplementary Figure 13

GATING STRATEGY FOR FLOW CYTOMETRIC DETECTION OF CXCR4 IN MMTV-NEU-DERIVED TUMORS

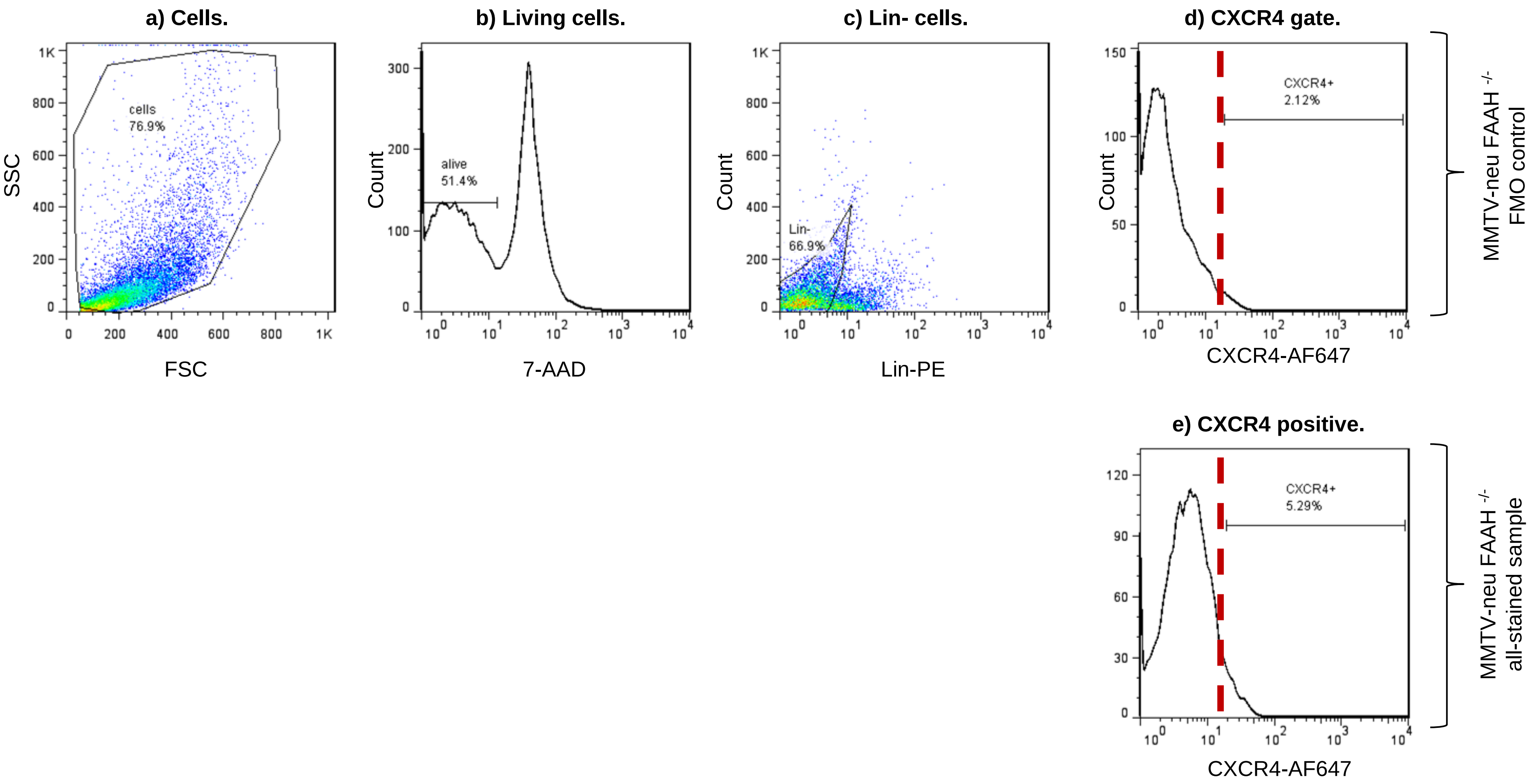

Supplementary Figure 13. Gating strategy used to determine the % of CXCR4 positivity in breast tumors derived from MMTV-neu mice. Gating strategy was always performed in fluorescence-minus-one (FMO) controls, which are samples stained with all the fluorophores except for CXCR4. There was a specific FMO control for each sample. **a** First, cells were separated from cell debris based on their size (FSC) and granularity (SSC). **b** 7-amino actinomycin D (7-AAD) was used as the live/dead discriminator. 7-AAD positive (dead) cells were excluded from the analysis. **c** CD31+, CD45+ and TER-119+ cells (all together labelled as "Lin+" cells) were excluded from the analysis. Positivity for Lin+ cells was set using its own FMO (not shown in the picture). **d** The fluorescent signal of 7-AAD-, Lin- cells in the FMO was used to set the upper boundary for background signal and thus to identify and gate positive populations for CXCR4. **e** The gating strategy described in a-d was applied to the all-stained sample to establish the % of CXCR4 positive cells.

Supplementary Data 1. Complete list of genes included in the RT^2 Profiler PCR array of mouse BC represented in Fig. 3h. Gene expression in MMTV-neu:$FAAH^{-/-}$ -derived tumors is depicted as log_2 fold change *vs*. MMTV-neu:$FAAH^{+/+}$ -derived tumors.

Supplementary Data 2. RNA-seq data showing differentially expressed genes upon FAAH silencing in T-47D cells, represented in Fig. 5a-c.

Supplementary Data 3. Complete list of genes included in the RT^2 Profiler PCR array of human tumor metastasis represented in Fig. 5e. Gene expression in FAAH-overexpressing MDA-MB-231 cells is depicted as log_2 fold change *vs*. parental cells.
