# Supplementary material for: Identification of fatty acid amide hydrolase as a metastasis suppressor in breast cancer
Source: Nat Commun. 2023 May 30;14:3130. doi: 10.1038/s41467-023-38750-9 (PMC10229544; doi:10.1038/s41467-023-38750-9)
Supplement: Supplementary file 3 — Reporting Summary [file 41467_2023_38750_MOESM3_ESM.pdf]

nature portfolio

Corresponding author(s): Eduardo Pérez-Gómez and Cristina Sánchez

Last updated by author(s): Feb 20, 2023

Reporting Summary

Nature Portfolio wishes to improve the reproducibility of the work that we publish. This form provides structure for consistency and transparency in reporting. For further information on Nature Portfolio policies, see our Editorial Policies and the Editorial Policy Checklist.

Statistics

For all statistical analyses, confirm that the following items are present in the figure legend, table legend, main text, or Methods section.

n/a	Confirmed	
☐	☒	The exact sample size (*n*) for each experimental group/condition, given as a discrete number and unit of measurement
☐	☒	A statement on whether measurements were taken from distinct samples or whether the same sample was measured repeatedly
☐	☒	The statistical test(s) used AND whether they are one- or two-sided<br>*Only common tests should be described solely by name; describe more complex techniques in the Methods section.*
☐	☒	A description of all covariates tested
☐	☒	A description of any assumptions or corrections, such as tests of normality and adjustment for multiple comparisons
☐	☒	A full description of the statistical parameters including central tendency (e.g. means) or other basic estimates (e.g. regression coefficient) AND variation (e.g. standard deviation) or associated estimates of uncertainty (e.g. confidence intervals)
☐	☒	For null hypothesis testing, the test statistic (e.g. *F*, *t*, *r*) with confidence intervals, effect sizes, degrees of freedom and *P* value noted<br>*Give P values as exact values whenever suitable.*
☒	☐	For Bayesian analysis, information on the choice of priors and Markov chain Monte Carlo settings
☒	☐	For hierarchical and complex designs, identification of the appropriate level for tests and full reporting of outcomes
☐	☒	Estimates of effect sizes (e.g. Cohen's *d*, Pearson's *r*), indicating how they were calculated

Our web collection on statistics for biologists contains articles on many of the points above.

Software and code

Policy information about availability of computer code

Data collection	- Fluorescence confocal images were acquired by using an Olympus FV1200 microscope.<br>- qPCR data were acquired with a QuantStudio™ 12K Flex system (Applied Biosystems).<br>- Flow cytometry data were acquired with a FACSCalibur system (Becton Dickinson).<br>- Bioluminescence data were acquired with an IVIS Spectrum system (Perkin Elmer).
Data analysis	Statistical analysis of the collected numerical data was routinely performed with IBM SPSS Statistics 28.0.1.1 and GraphPad Prism version 8.0.1 (GraphPad Software).<br><br>In addition:<br><br>- Confocal fluorescence was analyzed with Fiji ImageJ open-source software, version 1.53 (NIH).<br>- Flow cytometry data were analyzed with FlowJo™ software, version 10.6.2 (Becton, Dickinson and Company, 2019).<br>- Bioluminescence data were analyzed with with Living Image® software, version 4.7.4 (Perkin Elmer).<br>- Western blot experiments were analyzed with ImageJ open-source software version 1.52n (NIH).<br>- GSEA analysis was permormed with GSEA v4.3.2 (Broad Institute, Inc., Massachusetts Institute of Technology, and Regents of the University of California).

For manuscripts utilizing custom algorithms or software that are central to the research but not yet described in published literature, software must be made available to editors and reviewers. We strongly encourage code deposition in a community repository (e.g. GitHub). See the Nature Portfolio guidelines for submitting code & software for further information.

Data

Policy information about availability of data

All manuscripts must include a data availability statement. This statement should provide the following information, where applicable:

- Accession codes, unique identifiers, or web links for publicly available datasets
- A description of any restrictions on data availability
- For clinical datasets or third party data, please ensure that the statement adheres to our policy

The raw sequence data generated in this study is available at the Gene Expression Omnibus database under the following accession numbers: GSE197984 (https://www.ncbi.nlm.nih.gov/geo/query/acc.cgi?acc=GSE197984).

The following databases/datasets were available from the following links:
TCGA and Metabric database (http://www.cbioportal.org)
bc-GenExMiner database (https://www.institut-cancerologie-ouest.com)
E-TABM-158 (https://www.ebi.ac.uk/biostudies/arrayexpress/studies/E-TABM-158)
NKI_295 (https://www.nejm.org/doi/full/10.1056/nejmoa021967)
GEO DataSets :GSE27473; GSE2034; GSE69017; GSE7515 (https://www.ncbi.nlm.nih.gov/geo)
Minn et al. PNAS 2007 (https://www.pnas.org/doi/10.1073/pnas.0701138104)
CHEA Transcription Factor Targets dataset (https://maayanlab.cloud/Harmonizome)

GSEA molecular signatures were downloaded from the following database: https://www.gsea-msigdb.org/gsea/index.jsp.
CHARAFE_BREAST_CANCER_LUMINAL_VS_BASAL
CHARAFE_BREAST_CANCER_BASAL_VS_MESENCHYMAL
LIM_MAMMARY_STEM_CELL
PID_CXCR4_PATHWAY
VANTVEER_BREAST_CANCER_METASTASIS
SARRIO_EPITHELIAL_MESENCHYMAL_TRANSITION
LUNGMETASTASIS

Human research participants

Policy information about studies involving human research participants and Sex and Gender in Research.

Reporting on sex and gender	All patients were female and their gender was not considered in this study. Any human data displayed in the manuscript has been published before (Wenners AS et al. (2012). PLoS ONE 7(10): e45826. and Orea-Soufi A et al. (2021) Cancers , 13(21), 5307). Information regarding recruitment, ethics and population characteristics is reported in the original publications.
Population characteristics	Female breast cancer patients ranging from 24 to 78 years.<br>TMA #1 included 617 breast tumor samples of all BC subtypes from cases operated in the University Hospitals of Kiel, Tübingen, or Freiburg between 1997 and 2010.<br>TMA #2 included 276 pair-matched samples of luminal BC and affected lymph nodes from cases operated in the University Hospital of Donostia and Onkologikoa between 2001 and 2005.<br>For AEA measurement, a series of 37 breast tumors of all BC subtypes from the tumor biobank of University Hospital of Hierro was used.
Recruitment	No patients were recruited for these analyses.
Ethics oversight	All patients gave informed consent, and the study was authorized by the respective Hospital Ethics Committees (University Hospitals of Kiel, Tübingen, or Freiburg, University Hospital of Donostia and Onkologikoa and Hospital Universitario Puerta de Hierro).

Note that full information on the approval of the study protocol must also be provided in the manuscript.

Field-specific reporting

Please select the one below that is the best fit for your research. If you are not sure, read the appropriate sections before making your selection.

☒ Life sciences ☐ Behavioural & social sciences ☐ Ecological, evolutionary & environmental sciences

For a reference copy of the document with all sections, see nature.com/documents/nr-reporting-summary-flat.pdf

Life sciences study design

All studies must disclose on these points even when the disclosure is negative.

Sample size	The sample size for each experiment was estimated on the basis of previous studies conducted by our laboratories using similar in vitro, ex vivo and in vivo models. Power analysis, when necessary, was conducted with IBM SPSS software (IBM).
Data exclusions	No data were excluded for the statistical analyses.
Replication	The number of biological replicates (e.g., number of mice, number of experiments, number of cell cultures, number of cells) is provided in the corresponding figures/legends.
Randomization	In all experiments, biological samples (cultured cells and tissue extracts) and animals (mice) were allocated randomly into the different groups.
Blinding	All experiments were performed and analyzed in a blinded manner for mouse genotype and pharmacological treatment.

Reporting for specific materials, systems and methods

We require information from authors about some types of materials, experimental systems and methods used in many studies. Here, indicate whether each material, system or method listed is relevant to your study. If you are not sure if a list item applies to your research, read the appropriate section before selecting a response.

Materials & experimental systems

n/a	Involved in the study	
☐	☒	Antibodies
☐	☒	Eukaryotic cell lines
☒	☐	Palaeontology and archaeology
☐	☒	Animals and other organisms
☒	☐	Clinical data
☒	☐	Dual use research of concern

Methods

n/a	Involved in the study	
☒	☐	ChIP-seq
☐	☒	Flow cytometry
☒	☐	MRI-based neuroimaging

Antibodies

Antibodies used

The following antibodies were used throughout this study (company, ref, dilution use):
- FAAH (Abcam #ab128917, [EPR7549], dilution 1:1000)
- CXCR4 (Abcam #124824, [UMB2], dilution 1:500)
- β-Actin (Sigma-Aldrich #A5441, (H-6), dilution 1:5000)
- CK8 (DSHB #531826, TROMA-I, dilution 1:50)
- CK14 (Abcam #ab181595, [EPR17350], dilution 1:50)
- PCNA (EMD Millipore, MAB424R , clone PC10, dilution 1:10.000)
- CXCR4 (R&D Systems #MAB173, Clone # 44717, dilution 1:20)
- Biotinylated CD31 (Thermo Fisher Scientific #13-0311-82, clone 390, dilution 1:200)
- Biotinylated CD45 (Thermo Fisher Scientific #13-0451-82, clone 30-F11, dilution 1:400)
- Biotinylated TER119 (Thermo Fisher Scientific #13-5921-82, clone TER-119, dilution 1:100)
- Alexa Fluor™ 647 Phalloidin (Thermo Fisher Scientific #A22287, dilution 1:40)
- Alexa Fluor™ 647 mCXCR4 (Biolegend #146503, clone L276F12, dilution 1:100)
- E-cadherin (Cell Signaling Technology #3195, clone 24E10, dilution 1:200)
- Vimentin (BD Biosciences #550513, clone RV202, dilution 1:200)

Validation

All antibodies were used in this study according to manufacturer's instructions and after at least preliminary testing in our laboratories. Precise information on the validation of each primary antibody for species and applications can be found in the following links:
- FAAH (Abcam #ab128917, [EPR7549], dilution 1:1000)
https://www.abcam.com/products/primary-antibodies/faah1-antibody-epr7549-ab128917.html
- CXCR4 (Abcam #124824, [UMB2], dilution 1:500)
https://www.abcam.com/products/primary-antibodies/cxcr4-antibody-umb2-ab124824.html
- β-Actin (Sigma-Aldrich #A5441, (H-6), dilution 1:5000)
https://www.sigmaaldrich.com/ES/es/product/sigma/a5441
- CK8 (DSHB #531826, TROMA-I, dilution 1:50)
https://dshb.biology.uiowa.edu/TROMA-I
- CK14 (Abcam #ab181595, [EPR17350], dilution 1:50)
https://www.abcam.com/products/primary-antibodies/cytokeratin-14-antibody-epr17350-cytoskeleton-marker-ab181595.html
- PCNA (EMD Millipore, MAB424R , clone PC10, dilution 1:10.000)

https://www.merckmillipore.com/ES/es/product/Anti-PCNA-Antibody-clone-PC10,MM_NF-MAB424R
- CXCR4 (R&D Systems #MAB173, Clone # 44717, dilution 1:20)
https://www.rndsystems.com/products/human-cxcr4-antibody-44717_mab173
- Biotinylated CD31 (Thermo Fisher Scientific #13-0311-82, clone 390, dilution 1:200)
https://www.thermofisher.com/antibody/product/CD31-PECAM-1-Antibody-clone-390-Monoclonal/13-0311-82
- Biotinylated CD45 (Thermo Fisher Scientific #13-0451-82, clone 30-F11, dilution 1:400)
https://www.thermofisher.com/antibody/product/CD45-Antibody-clone-30-F11-Monoclonal/13-0451-82
- Biotinylated TER119 (Thermo Fisher Scientific #13-5921-82, clone TER-119, dilution 1:100)
https://www.thermofisher.com/antibody/product/TER-119-Antibody-clone-TER-119-Monoclonal/13-5921-82
- Alexa Fluor™ 647 Phalloidin (Thermo Fisher Scientific #A22287, dilution 1:40)
https://www.thermofisher.com/order/catalog/product/A22287
- Alexa Fluor™ 647 mCXCR4 (Biolegend #146503, clone L276F12, dilution 1:100)
https://www.biolegend.com/en-us/products/alexa-fluor-647-anti-mouse-cd184-cxcr4-antibody-8913
- E-cadherin (Cell Signaling Technology #3195, clone 24E10, dilution 1:200)
https://www.cellsignal.com/products/primary-antibodies/e-cadherin-24e10-rabbit-mab/3195
- Vimentin (BD Biosciences #550513, clone RV202, dilution 1:200)
https://www.bdbiosciences.com/en-us/products/reagents/microscopy-imaging-reagents/purified-mouse-anti-vimentin.550513

Eukaryotic cell lines

Policy information about cell lines and Sex and Gender in Research

Cell line source(s)	T-47D and MDA-MB-231 cell lines were purchased from the American Type Culture Collection (ATCC).
Authentication	T-47D and MDA-MB-231 cell lines were authenticated by analyzing STRs (microsatellites) from specific loci within the Human Genome at the Genomics Service of the Instituto de Investigaciones Biomédicas "Alberto Sols" (CSIC-UAM), Madrid, Spain
Mycoplasma contamination	T-47D and MDA-MB-231 cell lines were negative for mycoplasma contamination.
Commonly misidentified lines (See ICLAC register)	No misidentified cell lines were used in the study.

Animals and other research organisms

Policy information about studies involving animals; ARRIVE guidelines recommended for reporting animal research, and Sex and Gender in Research

Laboratory animals	Species: Mus musculus; strain: FVB/NJ and C57BL/6J age: 6 wk - 1 year. MMTV-neu mice (The Jackson Laboratory, Bar Harbor, ME, EE.UU.) and FAAH-/- mice (kind donation by Dr. Ben Cravatt's laboratory, Scripps Research Institute, La Jolla, CA, US) were used to generate of the congenic strain MMTV-neu:FAAH -/- all of them generated in FVB/NJ background.
Wild animals	The study did not involve wild animals.
Reporting on sex	Only female mice have been used for this study due to the nature of the tumor studied.
Field-collected samples	The study did not involve samples collected from the field.
Ethics oversight	Experimental procedures were performed in accordance with the guidelines and approval of the Animal Welfare Committees of Universidad Complutense de Madrid, and Comunidad de Madrid, the directives of the Spanish Government and the European Commission with approval reference numbers PROEX_126/15 and PROEX 183_19.

Note that full information on the approval of the study protocol must also be provided in the manuscript.

Flow Cytometry

Plots

Confirm that:

☒ The axis labels state the marker and fluorochrome used (e.g. CD4-FITC).

☒ The axis scales are clearly visible. Include numbers along axes only for bottom left plot of group (a 'group' is an analysis of identical markers).

☒ All plots are contour plots with outliers or pseudocolor plots.

☒ A numerical value for number of cells or percentage (with statistics) is provided.

Methodology

Sample preparation	Adherent cell lines were harvested using TrypLE™ Express (Gibco), washed with FACS buffer (PBS + 1 % FBS + 1 % BSA + 0.02 % sodium azide) and incubated with hCXCR4 primary antibody (R&D Systems #MAB173, dilution 1:20) and then Alexa Fluor™

	647 Mouse IgG (Invitrogen #A-21235, dilution 1:200). 7-aminoactinomycin D (7-AAD) (Biolegend, dilution 1:100) was used as a cell death marker and added 15' before the analysis. In the case of breast tumors derived from MMTV-neu mice, a single cell solution was obtained from tumors after mechanical disaggregation followed by 2 h of enzymatic digestion in DMEM + 125 µg/mL collagenase (Sigma-Aldrich) at 37 °C. CXCR4 staining was performed as described above with already conjugated primary antibody Alexa Fluor™ 647 mCXCR4 (Biolegend #146503, dilution 1:100). Cell suspension was also incubated with biotinylated CD31 (Thermo Fisher Scientific #13-0311-82, dilution 1:200), CD45 (Thermo Fisher Scientific #13-0451-82, dilution 1:400) and TER119 (Thermo Fisher Scientific #13-5921-82, dilution 1:100) + PE Streptavidin (Biolegend #405203, dilution 1:300) to exclude endothelial and hematopoietic lineages (Lin+ cells) from the analysis.
Instrument	FACSCalibur system (Becton Dickinson).
Software	FlowJo™ software (Becton, Dickinson and Company, 2019).
Cell population abundance	Analysis of CXCR4+ cells from breast cancer cell lines and breast tumors was performed after exclusion of dead (7-AAD+) cells (which usually ranged around 5 % of total cells for cell lines and between 20 and 50 % for breast tumors) and, in the case of breast tumors, Lin+ cells (which usually ranged between 10 and 30 % of 7-AAD- cells). CXCR4+ cells usually ranged between 5 and 30 % of 7-AAD-, Lin - cells.
Gating strategy	Gating strategy was always performed in fluorescence-minus-one (FMO) controls, which are samples stained with all the fluorophores except for CXCR4. There was a specific FMO control for each sample. For breast cancer cell lines, cells were separated from cell debris based on their size (FSC) and granularity (SSC). Then, 7-AAD was used as the live/dead discriminator. 7-AAD+ (dead) cells were excluded from the analysis. The fluorescent signal of the remaining (7-AAD- alive) cells in the FMO was used to set the upper boundary for background signal and thus to identify and gate positive populations for CXCR4. In the case of breast tumors, the gating strategy was performed as described above but including an extra step after dead cell exclusion to exclude Lin+ (CD31+, CD45+ and TER-119+) cells.

☒ Tick this box to confirm that a figure exemplifying the gating strategy is provided in the Supplementary Information.
