# Supplementary material for: Identification of fatty acid amide hydrolase as a metastasis suppressor in breast cancer
Source: Nat Commun. 2023 May 30;14:3130. doi: 10.1038/s41467-023-38750-9 (PMC10229544; doi:10.1038/s41467-023-38750-9)
Supplement: Supplementary file 4 — Description of Additional Supplementary Files [file 41467_2023_38750_MOESM4_ESM.docx]

**Description of Additional Supplementary Files**

Supplementary Data 1:

Description: Complete list of genes included in the RT^2^ Profiler PCR array of mouse BC represented in Fig. 3h. Gene expression in MMTV-neu:FAAH^-/-^ -derived tumors is depicted as log^2^ fold change *vs.* MMTV-neu:FAAH^+/+^ -derived tumors

Supplementary Data 2:

Description: RNA-seq data showing differentially expressed genes upon FAAH silencing in T-47D cells, represented in Fig. 5a-c.

Supplementary Data 3:

Description: Complete list of genes included in the RT^2^ Profiler PCR array of human tumor metastasis represented in Fig. 5e. Gene expression in FAAHoverexpressing MDA-MB-231 cells is depicted as log2 fold change *vs.* parental cells.
